# Influence of Safety Warnings on the Prescribing Attitude of JAK Inhibitors for Rheumatoid Arthritis in Italy

**DOI:** 10.3390/jcm13133929

**Published:** 2024-07-04

**Authors:** Marino Paroli, Andrea Becciolini, Alberto Lo Gullo, Simone Parisi, Elena Bravi, Romina Andracco, Valeria Nucera, Francesca Ometto, Federica Lumetti, Antonella Farina, Patrizia Del Medico, Matteo Colina, Viviana Ravagnani, Palma Scolieri, Maddalena Larosa, Marta Priora, Elisa Visalli, Olga Addimanda, Rosetta Vitetta, Alessandro Volpe, Alessandra Bezzi, Francesco Girelli, Aldo Biagio Molica Colella, Rosalba Caccavale, Eleonora Di Donato, Giuditta Adorni, Daniele Santilli, Gianluca Lucchini, Eugenio Arrigoni, Ilaria Platè, Natalia Mansueto, Aurora Ianniello, Enrico Fusaro, Maria Chiara Ditto, Vincenzo Bruzzese, Dario Camellino, Gerolamo Bianchi, Francesca Serale, Rosario Foti, Giorgio Amato, Francesco De Lucia, Ylenia Dal Bosco, Roberta Foti, Massimo Reta, Alessia Fiorenza, Guido Rovera, Antonio Marchetta, Maria Cristina Focherini, Fabio Mascella, Simone Bernardi, Gilda Sandri, Dilia Giuggioli, Carlo Salvarani, Maria Ilenia De Andres, Veronica Franchina, Francesco Molica Colella, Giulio Ferrero, Bernd Raffeiner, Alarico Ariani

**Affiliations:** 1 Department of Clinical, Internist, Anesthesiologic and Cardiovascular Sciences, Sapienza University of Rome, 00185 Rome, Italy; rosalba_caccavale@yahoo.it; 2 Internal Medicine and Rheumatology Unit, University Hospital of Parma, 43126 Parma, Italy; beccio@yahoo.it (A.B.); eleonoradidonato@ymail.com (E.D.D.); gadorni@ao.pr.it (G.A.); dsantilli@ao.pr.it (D.S.); glucchini@ao.pr.it (G.L.); dott.alaricoariani@libero.it (A.A.); 3 Rheumatology Unit, ARNAS Garibaldi, 95124 Catania, Italy; albertologullo@virgilio.it (A.L.G.); ilenia.deandres@gmail.com (M.I.D.A.); 4 Rheumatology Department, Azienda Ospedaliero-Universitaria Città della Salute e della Scienza di Torino, 10126 Turin, Italy; simone.parisi@hotmail.it (S.P.); fusaro.reumatorino@gmail.com (E.F.);; 5 Rheumatology Unit, Guglielmo da Saliceto Hospital, 29121 Piacenza, Italy; e.bravi@ausl.pc.it (E.B.); e.arrigoni@ausl.pc.it (E.A.); i.plate@ausl.pc.it (I.P.); 6 Rheumatology Unit, ASL1 Liguria, 18038 Bussana di Sanremo, Italy; r.andracco@gmail.com (R.A.); natalia.mansueto@libero.it (N.M.); 7 Rheumatology Unit, ASL Novara, 28100 Novara, Italy; v.nucera@asl.novara.it (V.N.); a.ianniello@asl.novara.it (A.I.); 8 Rheumatology Unit, Azienda ULSS 6 Euganea, 35131 Padua, Italy; f.ometto@gmail.com; 9 Rheumatology Unit, Azienda USL of Modena and AOU Policlinico of Modena, 41100 Modena, Italy; fedelumetti@gmail.com; 10 Internal Medicine Unit, Augusto Murri Hospital, 63900 Fermo, Italy; antonella_farina@hotmail.com; 11 Internal Medicine Unit, Civitanova Marche Hospital, 62012 Civitanova Marche, Italy; patdelmedico@hotmail.com; 12 Rheumatology Unit, Internal Medicine Division, Department of Medicine and Oncology, Santa Maria della Scaletta Hospital, 40026 Imola, Italy; matteo.colina2@unibo.it; 13 Rheumatology Unit, Alma Mater Studiorum—University of Bologna, 40126 Bologna, Italy; 14 Rheumatology Unit, Santa Chiara Hospital APSS—Trento, 38122 Trento, Italy; viviana.ravagnani@gmail.com; 15 Rheumatology Unit, Nuovo Regina Margherita Hospital, 00154 Rome, Italy; palma.scolieri@gmail.com (P.S.); vinbruzzese@tiscali.it (V.B.); 16 Division of Rheumatology, Department of Medical Specialties, Azienda Sanitaria Locale 3 Genovese, 16132 Genoa, Italy; madlarosa8@gmail.com (M.L.); dario.camellino@asl3.liguria.it (D.C.); gerolamo.bianchi@asl3.liguria.it (G.B.); 17 Rheumatology Unit, ASL CN1, 12100 Cuneo, Italy; marta.priora@gmail.com (M.P.); francesca.serale@gmail.com (F.S.); 18 Rheumatology Unit, Policlinico San Marco Hospital, 95121 Catania, Italy; elivisa21@gmail.com (E.V.); rosfoti5@gmail.com (R.F.); giorgioamato@hotmail.it (G.A.); francescodelucia89@yahoo.it (F.D.L.); yleniadalbosco@gmail.com (Y.D.B.); robertafoti@hotmail.com (R.F.); 19 Rheumatology Unit, AUSL of Bologna—Policlinico Sant’Orsola—AOU—IRCCS of Bologna, 40138 Bologna, Italy; olga.addimanda@ausl.bologna.it (O.A.); massimo.reta@ausl.bologna.it (M.R.); 20 Rheumatology Unit, ASL VC Sant’Andrea Hospital, 13100 Vercelli, Italy; rosetta.vitetta@aslvc.piemonte.it (R.V.); alessia.fiorenza@aslvc.piemonte.it (A.F.); guido.rovera.gr@gmail.com (G.R.); 21 Rheumatology Unit, IRCCS Sacro Cuore Don Calabria Hospital, 37024 Negrar di Valpolicella, Italy; avolpe127@gmail.com (A.V.); antonio.marchetta@sacrocuore.it (A.M.); 22 Internal Medicine and Rheumatology Unit, AUSL della Romagna—Rimini, 47924 Rimini, Italy; alessandra.bezzi@auslromagna.it (A.B.); mariacristina.focherini@auslromagna.it (M.C.F.); fabio.mascella@auslromagna.it (F.M.); 23 Rheumatology Unit, G.B. Morgagni—L. Pierantoni Hospital, 47121 Forlì, Italy; francesco.girelli@auslromagna.it (F.G.); siiberna@yahoo.it (S.B.); 24 Departmental Unit of Rheumatology, Azienda Ospedaliera Papardo, 98158 Messina, Italy; aldomolica@alice.it; 25 Rheumatology Unit, University of Modena and Reggio Emilia, 41125 Modena, Italy; gilda.sandri@unimore.it (G.S.); dilia.giuggioli@unimore.it (D.G.); carlo.salvarani@unimore.it (C.S.); 26 Medical Oncology Unit, Azienda Ospedaliera Papardo, 98158 Messina, Italy; verifra82@yahoo.it; 27 Medicine Unit, Milano-Bicocca University, 20126 Milan, Italy; francesco.molica3@gmail.com; 28 Unit of Diagnostic and Interventional Radiology, Santa Corona Hospital, 17027 Pietra Ligure, Italy; giulio.ferrero@gmail.com; 29 Department of Rheumatology, Bolzano Central Hospital, 39100 Bolzano, Italy; berndraffeiner@yahoo.com

**Keywords:** janus kinase inhibitors, safety warnings, prescribing attitude, rheumatoid arthritis

## Abstract

**Background/Objectives:** The Janus kinase inhibitors (JAKi) tofacitinib (TOFA), baricitinib (BARI), upadacitinib (UPA), and filgotinib (FILGO) are effective drugs for the treatment of rheumatoid arthritis. However, the US Food and Drug Administration (FDA) raised concerns about the safety of TOFA after its approval. This prompted the European Medicines Agency (EMA) to issue two safety warnings for limiting TOFA use, then extended a third warning to all JAKi in patients at high risk of developing serious adverse effects (SAE). These include thrombosis, major adverse cardiac events (MACE), and cancer. The purpose of this work was to analyze how the first two safety warnings from the EMA affected the prescribing of JAKi by rheumatologists in Italy. **Methods:** All patients with rheumatoid arthritis who had been prescribed JAKi for the first time in a 36-month period from 1 July 2019, to 30 June 2022 were considered. Data were obtained from the medical records of 29 Italian tertiary referral rheumatology centers. Patients were divided into three groups of 4 months each, depending on whether the JAKi prescription had occurred before the EMA’s first safety alert (1 July–31 October 2019, Group 1), between the first and second alerts (1 November 2019–29 February 2020, Group 2), or between the second and third alerts (1 March 2021–30 June 2021, Group 3). The percentages and absolute changes in the patients prescribed the individual JAKi were analyzed. Differences among the three groups of patients regarding demographic and clinical characteristics were also assessed. **Results:** A total of 864 patients were prescribed a JAKi during the entire period considered. Of these, 343 were identified in Group 1, 233 in Group 2, and 288 in Group 3. An absolute reduction of 32% was observed in the number of patients prescribed a JAKi between Group 1 and Group 2 and 16% between Group 1 and Group 3. In contrast, there was a 19% increase in the prescription of a JAKi in patients between Group 2 and Group 3. In the first group, BARI was the most prescribed drug (227 prescriptions, 66.2% of the total), followed by TOFA (115, 33.5%) and UPA (1, 0.3%). In the second group, the most prescribed JAKi was BARI (147, 63.1%), followed by TOFA (65, 27.9%) and UPA (33, 11.5%). In the third group, BARI was still the most prescribed JAKi (104 prescriptions, 36.1%), followed by UPA (89, 30.9%), FILGO (89, 21.5%), and TOFA (33, 11.5%). The number of patients prescribed TOFA decreased significantly between Group 1 and Group 2 and between Group 2 and Group 3 (*p* ˂ 0.01). The number of patients who were prescribed BARI decreased significantly between Group 1 and Group 2 and between Group 2 and Group 3 (*p* ˂ 0.01). In contrast, the number of patients prescribed UPA increased between Group 2 and Group 3 (*p* ˂ 0.01). **Conclusions**: These data suggest that the warnings issued for TOFA were followed by a reduction in total JAKi prescriptions. However, the more selective JAKi (UPA and FILGO) were perceived by prescribers as favorable in terms of the risk/benefit ratio, and their use gradually increased at the expense of the other molecules.

## 1. Introduction

Rheumatoid arthritis (RA) is a chronic autoimmune disease characterized by systemic inflammation and persistent synovitis, which can lead to joint damage, disability, and significant comorbidities [1]. The pathogenesis of RA involves a complex interaction between genetic, environmental, and immunological factors that lead to the activation of the immune system against joint tissues [2]. The latest recommendations of the European Alliance of Associations for Rheumatology (EULAR) recommend adding or switching to biologic disease-modifying antirheumatic drugs (bDMARDs) or targeted synthetic DMARDs (tsDMARDs), such as JAK inhibitors (JAKi), for patients with inadequate responses to conventional synthetic DMARDs (csDMARDs) [3]. Specifically, JAKi are a class of drugs approved in recent years for the treatment of rheumatoid arthritis [4] that inhibit the JAK enzymes involved in cytokine signaling through the intracellular JAK/STAT pathway, leading in turn to the activation of intranuclear transcription factors of several genes encoding for pro-inflammatory molecules [5]. Currently, four JAKi have been approved by the European Medicines Agency (EMA). These include tofacitinib (TOFA), which mainly inhibits JAK1 and JAK2 and to a lesser extent JAK2; baricitinib (BARI), which primarily inhibits JAK 1 and JAK2; and upadacitinib (UPA) and filgotinib (FILGO), both selective for JAK1 [6]. In addition to the indication for the treatment of rheumatoid arthritis, TOFA and UPA have been approved for the treatment of psoriatic arthritis (PsA) and radiographic (TOFA and UPA) or non-radiographic (UPA) axial spondyloarthritis (axSpA) [7]. JAKi have some advantages over biologics, such as oral administration, rapid onset of action, broad mechanism of action, efficacy in treatment-resistant cases, and absence of immunogenicity, which, on the other hand, may be responsible for the formation of anti-drug antibodies (ADAs) during biologic therapy, possibly reducing its efficacy [8]. Although in registrational studies these drugs have been shown to have an acceptable safety profile, further concerns emerged in the post-marketing phase for TOFA regarding the possible increased risk of thromboembolism, major cardiovascular events (MACE), and cancer development [9,10,11]. This prompted the FDA to add in July of 2019 a warning (Boxed Warning) to the TOFA label and to request a phase 3b-4, a randomized, open-label, noninferiority ORAL surveillance study [12,13]. In November of that year, the EMA issued a similar notice, followed by two subsequent notices in March of 2021 and November of 2022 [14] emphasizing the increased risk of the aforementioned complications and serious and fatal infections associated with TOFA use. Therefore, in accordance with EMA recommendations, the manufacturer informed the physicians of TOFA-associated risks, recommending that a dose of 5 mg twice daily for the treatment of rheumatoid arthritis not be exceeded and treating patients older than 65 years only when alternative treatments are not available. Moreover, physicians were made aware that patients aged 50 years and older with at least one additional cardiovascular risk factor had been shown by ORAL surveillance study to have an increased incidence of myocardial infarction compared with patients treated with an anti-TNF-alpha biologic, and that there was in the treated patients an increased incidence of malignancies, including lung cancer and lymphoma. This communication was followed by a similar recommendation that TOFA should not be used in patients older than 65 years, in smokers or former smokers, and in patients with other risk factors for cardiovascular disease, unless suitable therapeutic alternatives are unavailable. In January of 2023, the EMA’s Committee for Medicinal Products for Human Use (CHMP) approved the measures recommended by the Pharmacovigilance Risk Assessment Committee (PRAC). These recommendations not only confirmed that TOFA increases the risk of major cardiovascular problems, cancer, VTE, serious infections, and death from any cause compared with drugs in the TNF-alpha inhibitor class but were also extended to all other officially approved JAKi, although specific studies on these molecules had not yet been conducted [15].

The purpose of the present multicenter study was to analyze any changes in the prescribing attitude of JAKi in the treatment of rheumatoid arthritis by Italian rheumatologists over a period of 36 months, including the period before and after the first and second EMA safety warnings.

## 2. Material and Methods

All patients with rheumatoid arthritis who had been prescribed a specific JAK inhibitor for the first time within a 36-month period from 1 July 2019 to 30 June 2022 were considered. The patients were then divided into three groups based on the timing of their prescriptions: Group 1 included patients prescribed the JAK inhibitor before the first EMA safety alert (1 July–31 October 2019), Group 2 included those prescribed between the first and second alerts (1 November 2019–29 February 2021), and Group 3 included patients prescribed between the second and third alerts (1 March 2021–30 June 2022). The percentage and absolute changes in patients prescribed a single JAKi were analyzed. Differences in the three groups of patients, with regard to the demographics and clinical characteristics of the treated patients, were also assessed. Therefore, the differences in the prescription of JAKi in the three groups considered were analyzed in both absolute and relative terms with respect to single molecules. Any differences in the 3 groups considered with regard to the demographic and clinical data of the treated patients were also considered. Specifically, the male/female ratio, the age of the treated patients, the presence or absence of a cigarette smoking habit, and associated comorbidities were analyzed. Other parameters such as the seropositivity/seronegativity of rheumatoid arthritis, disease activity, and previous or concomitant treatments were also analyzed. Prescription data were extracted from the medical records of 29 tertiary rheumatology centers distributed in different Italian regions. This study is part of the BIologics Retention Rate Assessment (BIRRA) project, which focuses on studying the effects of current therapy in rheumatic diseases. The study was conducted according to the principles of the Declaration of Helsinki and was approved by local ethics committees, the main one being the Ethics Committee of the Emilia Vasta Nord Area, protocol code 34.713, approved on 28 August 2019. The statistical analysis was conducted by the Fisher–Yates exact test for qualitative data and the Mann–Whitney U test and the Wilcoxon Signed Rank test for quantitative data. A *p* value less than or equal to 0.05 or 0.01 indicated a statistically significant result. The MedCalc program, version 12.5.0.0 (MedCalc software Ltd., Ostend, Belgium), was used for statistical analysis.

## 3. Results

**Figure 1:**
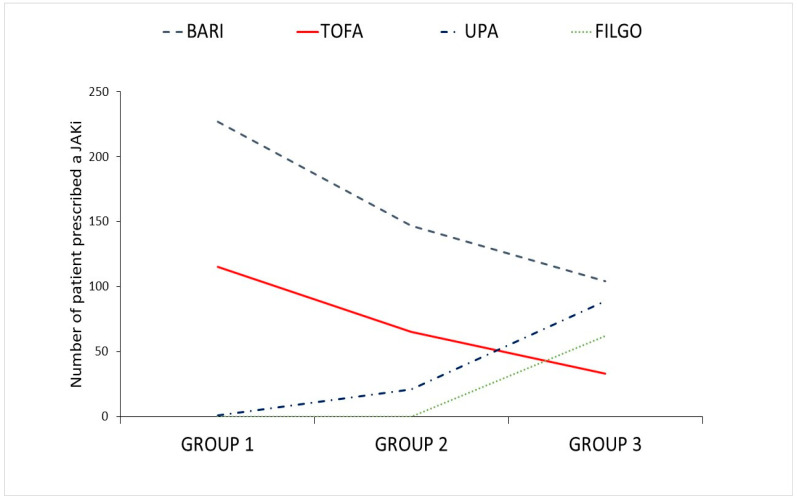
Variation over time in the number of patients prescribed a given JAKi.

A total of 864 patients were prescribed JAKi during the entire period considered in this study. Of these, 343 were identified in Group 1, 233 in Group 2, and 288 in Group 3. An absolute reduction of 32% was observed in the number of patients prescribed JAKi between Group 1 and Group 2 and 16% between Group 1 and Group 3. In contrast, there was a 19% increase of the prescription of JAKi in patients between Group 2 and Group 3. In the first group, BARI was the most prescribed drug (227 prescriptions, 66.2% of the total), followed by TOFA (115, 33.5%) and UPA (1, 0.3%). In the second group, the most prescribed JAKi was BARI (147, 63.1%), followed by TOFA (65, 27.9%) and UPA (33, 11.5%). In the third group, BARI was still the most prescribed JAKi (104 prescriptions, 36.1%), followed by UPA (89, 30.9%), FILGO (89, 21.5%), and TOFA (33, 11.5%). However, the number of patients prescribed TOFA decreased significantly between Group 1 and Group 2 and between Group 2 and Group 3 (*p* ˂ 0.01). The number of patients who were prescribed BARI decreased significantly between Group 1 and Group 2 and between Group 2 and Group 3 (*p* ˂ 0.01). In contrast, the number of patients prescribed UPA increased between Group 2 and Group 3 (*p* ˂ 0.01). The F:M ratio increased between Groups 1 and 2 (4.0 vs. 4.8; *p* ˂ 0.05) and then decreased between Groups 2 and 3 (4.8 vs. 2.7; *p* ˂ 0.01). A reduction in the visual analog scale of pain (VAS) 0–100 was observed in Group 2 as compared to Group 1 (median 60, IQR 40–80 vs median 70, IQR 50–80; *p* ˂ 0.05). There was also a significant reduction in dyslipidemia and cancer cases between Group 1 and Group 2 (*p* ˂ 0.05). Glucocorticoid use in combination with JAKi treatment increased significantly between Group 1 and Group 2 (*p* ˂ 0.05) and between Group 2 and Group 3 (*p* ˂ 0.01). All these data are summarized in Table 1 and Figure 1.

## 4. Discussion

In this multicenter retrospective observational study, we analyzed how the first two safety warnings issued by the EMA on the use of TOFA influenced the attitudes of Italian rheumatologists in prescribing TOFA and the other JAKi approved for rheumatoid arthritis.

The first observed finding is that the total number of patients prescribed JAKi decreased after the first and second safety alerts as compared to the previous period over a comparable time frame. Because we did not consider the total number of patients treated with other second-line drugs such as biologics during the periods covered by our study, we cannot demonstrate that the observed absolute reduction in the number of JAKi prescriptions is statistically significant. However, assuming with good probability that the total number of patients treated with any type of second-line drug was constant throughout the duration of the study, our results suggest that JAKI prescribing actually decreased after the safety alerts.

We found that the percentage of patients treated with the individual JAKi changed significantly over the different periods considered. Specifically, the relative percentage of prescriptions of non-selective JAKi and, in particular, the pan-JAK inhibitor TOFA and the selective JAK1/JAK2 inhibitor BARI, decreased significantly both in the comparison between Group 1 and Group 2 and in the comparison between Group 2 and Group 3.

In the pre-alert period, BARI was the most prescribed JAKi. This is probably due to some features of BARI, including its once-daily administration and the possibility of reducing the dosage in elderly subjects, making its use more flexible than TOFA [16]. BARI remained the most prescribed JAKi even in the later periods considered in this study, although its prescriptions decreased significantly along with those of TOFA despite the fact that the safety warning referred only to TOFA. This may be explained assuming that BARI was perceived to be just as risky as TOFA because it belongs to the same class of drugs.

On the other hand, we observed a gradual increase in prescriptions of the JAK1-selective inhibitor UPA throughout the period examined and a significant number of prescriptions of the JAK1-selective inhibitor FILGO in Group 3. It should be noted that UPA was approved and made reimbursable by the Italian Drug Agency (AIFA) after the start of the first study period considered. A portion of the UPA prescriptions in Group 1 were therefore allowed through a limited number of free samples provided by the manufacturer. Therefore, it was not possible to fairly compare UPA prescriptions in Group 1 with those in the subsequent groups. In addition, the statistical analysis of FILGO prescriptions was not possible because FILGO was approved only in the third period of our study.

It is likely that the reduction in prescriptions of non-selective or partially selective JAKi compared with second-generation JAK1-selective molecules was influenced by the perception that safety problems mainly affected older molecules. This probably favored the progressive prescriptions of UPA and FILGO over TOFA and BARI. It should be noted that this study was conducted prior to the EMA’s third warning of November 2022, which extended the alert to all JAKi molecules. However, data from another study indicate that even after this third alert, the prescriptions of JAK-1 selective inhibitors, and in particular UPA, continued to increase, even though selectivity is more a concept based on theory than on actual data [17].

The renal elimination of UPA has been shown to be minimal, with only about 20 percent of the dose eliminated in the urine as unchanged drug. This reduces the risk of drug accumulation in patients with impaired renal function, contributing to the safety of the treatment. Although data on the renal function of patients included in this study are not available, it cannot be ruled out that part of the increase in UPA prescriptions is due to this pharmacokinetic peculiarity of the drug [18]. In this regard, it has been reported that the co-occurrence of RA with end-stage renal disease (ESRD) is greater than 1%, probably due to the impact of diabetes and hypertension, comorbidities often associated with this rheumatic disease [19].

An unexpected result of our study was the relative decrease in the ratio of females to males treated with JAKi between Group 1 and Group 3. One possible explanation is the different risk perception of the therapy between the two genders, which consequently favored prescribing JAKi in males after receiving information about the potential risks from the physician [20]. A statistically significant reduction in cancer cases was also observed between Groups 1 and 2 (*p* ˂ 0.05). We do not have detailed information on the nature of the cancer cases reported during the study. However, all cases were incidental and led to the discontinuation of treatment with JAKi. Non-melanoma skin cancers (NMSCs) were excluded from the calculation. The reduction in cancer cases could be due to more careful patient selection following EMA warnings.

Finally, an interesting finding was the statistically significant increase in glucocorticoid use expressed as a prednisone-equivalent (PDN-eq) dose in combination with JAKi in Groups 2 and 3 as compared to the initial group (*p* ˂ 0.01), respectively. This could reflect an increasing confidence in the safety of JAKi due to actual experience in the use of these molecules, with a reduction in prescribers’ fear of possible cardiovascular and infectious adverse effects after combination with glucocorticoids [21,22]. In addition, the increased glucocorticoid use expressed as prednisone-equivalent doses in Groups 2 and 3 could be related to the higher severity of disease in these patients, who had already failed other therapies.

The limitations of the study are that the periods between the groups were not contiguous, and this could have introduced bias related to seasonal variations or other uncontrolled external factors. The periods were also not directly comparable because of different drug availability. In addition, the retrospective nature of the study could have introduced selection and information bias. An additional limitation is that the second period analyzed in this study coincided with the peak of COVID-19 cases. In this regard, a three-month observational study conducted in the United States showed that patients taking biologic DMARDs (bDMARDs) and JAKi were more likely to discontinue or delay drug use than those taking conventional synthetic DMARDs (csDMARDs). The main reason was concern about possible increased susceptibility or the severity of COVID-19, as well as the cancellation or postponement of appointments [23].

In conclusion, the data reported by this Italian multicenter study underscore that the first two EMA safety warnings negatively affected the use of JAKi but at the same time promoted a shift toward the use of more selective molecules in this class, and in particular the JAK1-selective molecules. Further studies are needed to clarify how JAKi prescribing might be affected in the future as more data on its safety become available. It will also be necessary to clarify whether safety issues will affect other indications of JAKi besides rheumatic diseases, such as chronic inflammatory bowel disease, psoriasis and atopic dermatitis. In this way, it will be possible to refine patient selection criteria, ensuring a balanced approach that maximizes therapeutic efficacy and minimizes potential adverse effects without depriving those who might benefit from JAKi therapy in view of the high disabling potential of immune-mediated inflammatory diseases.

## Figures and Tables

**Table 1:** Demographic and clinical characteristics of patients.

Characteristic		Group 1	Group 2	Group 3
N		343	233	288
M:F		68:275	40:193 *	78–210 *
Age, yrs [IQR]		61 54–70	58 48–66	58 50–67
BMI, kg/m^2^ [IQR]		24.6 22.7–27.0	24.7 23.0–27.0	25 22.8–27.9
Smokers, n yes:no:former		52:198:63	51:130:32	48:176:49
Disease duration, yrs [IQR]		6.3 1.8–13.5	6.5 3.2–12.8	6.7 2.6–12.8
RF presence, %		63.4	61.2	59.3
ACPA presence, %		59.5	58.4	54.9
Comorbidities, %	Diabetes Arterial hypertension Hypercholesterolemia MACE Cancer	9.3 37.0 26.5 5.2 6.4	5.2 30.9 18.0 3.4 2.1 *	9.0 35.7 21.9 6.6 5.2
Tender Joints, n [IQR]		8 4–12	8 4–12	8 4–12
Swollen Joints, n [IQR]		5 2–8	5 3–8	4 3–8
ESR, mm/h [IQR]		32 19–46	34 20–46	31 18–48
PCR, mg/dL [IQR]		1.3 0.5–3.0	1.3 0.6–2.6	1.2 0.5–3.8
VAS, 0–100 * [IQR]		60 40–80	70 50–80	70 50–80
PGA-Med, 0–10 [IQR]		7 5–8	5 5–8	7 5–7
DAS28 [IQR]		5.4 4.8–6.1	5.4 4.8–6.0	5.3 4.7–5.9
DAS28-CRP [IQR]		4.9 4.3–5.6	5.0 4.2–5.4	5.0 4.3–5.6
JAKi; %	Baricitinib Filgotinib Tofacitinib Upadacitinib	66.2 0 33.5 0.3	63.1 * 0 27.9 * 9.0	36.1 * 21.5 11.5 * 30.9 *
JAKi naive; %	None One Two	74.6 25.1 0.3	71.2 27.9 0.9	65.3 30.9 3.8
Treatment line after csDMARD-IR [IQR]		2 1–4	2 2–4	2 2–4
csDMARDs concomitant, %		49.0	45.5	43.4
Glucocorticoid *, %		32.7	42.0 *	43.8 *
Glucocorticoid, mg/day (PDN-eq) [IQR]		5 4–5	5 5–5	5 4–6

RF = rheumatoid factor; ACPA = anti-citrullinated peptide antibody; ESR = erythrocyte sedimentation rate; CRP = C-reactive protein; VAS = visual analog scale; PGA = global physical assessment; DAS = disease activity score; JAKi = JAK inhibitor; csDMARDs-IR = inadequate response to treatment with conventional synthetic DMARDs; PDN-eq = Prednisone-equivalent; * = statistically significant (see results sections for details).

## Data Availability

All data generated or analyzed during the study are included in this published article.
